# Exercise for the Diabetic Gut—Potential Health Effects and Underlying Mechanisms

**DOI:** 10.3390/nu14040813

**Published:** 2022-02-15

**Authors:** Sarah Valder, Christian Brinkmann

**Affiliations:** 1 Department of Molecular and Cellular Sport Medicine, Institute of Cardiovascular Research and Sport Medicine, German Sport University Cologne, 50933 Cologne, Germany; s.valder@dshs-koeln.de; 2 Department of Preventive and Rehabilitative Sport Medicine, Institute of Cardiovascular Research and Sport Medicine, German Sport University Cologne, 50933 Cologne, Germany; 3 Department of Fitness & Health, IST University of Applied Sciences, 40233 Dusseldorf, Germany

**Keywords:** exercise, gut microbiota, type 2 diabetes mellitus, gut barrier function, short-chain fatty acid

## Abstract

It can be assumed that changes in the gut microbiota play a crucial role in the development of type 2 diabetes mellitus (T2DM). It is generally accepted that regular physical activity is beneficial for the prevention and therapy of T2DM. Therefore, this review analyzes the effects of exercise training on the gut microbiota composition and the intestinal barrier function in T2DM. The current literature shows that regular exercise can influence the gut microbiota composition and the intestinal barrier function with ameliorative effects on T2DM. In particular, increases in the number of short-chain fatty acid (SCFA)-producing bacteria and improvements in the gut barrier integrity with reduced endotoxemia seem to be key points for positive interactions between gut health and T2DM, resulting in improvements in low-grade systemic inflammation status and glycemic control. However, not all aspects are known in detail and further studies are needed to further examine the efficacy of different training programs, the role of myokines, SCFA-producing bacteria, and SCFAs in the relevant metabolic pathways. As microbial signatures differ in individuals who respond differently to exercise training programs, one scientific focus could be the development of computer-based methods for the personalized analysis of the gut microbiota in the context of a microbiota/microbiome-based training program.

## 1. Introduction

Type 2 diabetes mellitus (T2DM) is one of the most relevant health issues of the 21st century. Due to the fact that diabetes is among the top 10 causes of deaths worldwide, it is of particular importance to find effective treatment and prevention measures to control the disease and its complications [1].

A healthy lifestyle is an important factor for successful T2DM management. Regular exercise training seems to be of particular significance. Studies have shown, for example, that exercise training can improve inflammation status, insulin sensitivity, and blood glucose control in T2DM patients [2,3,4]. Furthermore, weight loss in the context of a structured exercise program can facilitate the remission of T2DM [5].

**Figure 1:**
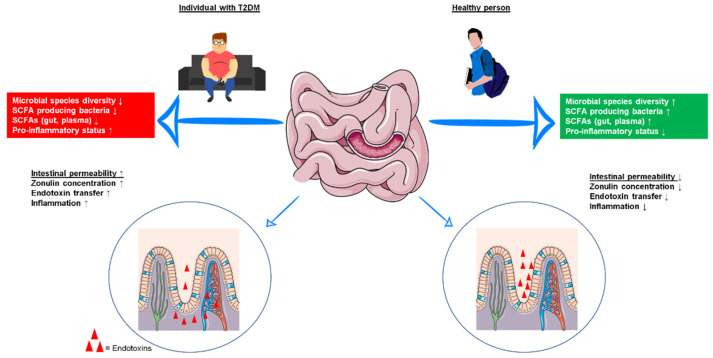
Microbiota composition and gut barrier function in individuals with type 2 diabetes mellitus (T2DM) and healthy persons. Patients with T2DM have lower microbial species diversity, lower levels of short-chain fatty acid (SCFA)-producing bacteria and lower levels of SCFAs in the gut and blood, which is accompanied by increased pro-inflammatory status. Furthermore, intestinal permeability is increased in T2DM. *SCFA* short-chain fatty acid, *T2DM* type 2 diabetes mellitus. Graphical sources: https://pixabay.com, https://smart.servier.com/smart_image/intestine-4/, https://smart.servier.com/smart_image/intestinal-villi (last access to all three websites: 2 July 2021).

Growing evidence suggests that the gut microbiota plays a crucial role in the development of T2DM. The human gut microbiota includes thousands of different bacterial species, as well as several archaea and viruses [6]. Together with more than three million genes and various influencing factors such as age, medication use, diet, and physical activity of the host, the gut microbiota represents a complex gut ecosystem that is very dynamic and highly individual [7,8,9,10,11]. An overview of the bacterial taxa described in this review can be found in Supplementary Material Figure S1. A balanced gut microbiota exerts a wide range of functions that positively affect the regulation of the intestinal mucosal barrier, the immune system through protection against pathogens, energy harvest, the fermentation of indigestible carbohydrates, or vitamin synthesis [12]. Disturbances in this balance of gut microbial colonization (e.g., through reduced intestinal bacterial diversity) are associated with enhanced lipid levels, increased inflammatory processes, and insulin resistance (IR), and more generally with an increased risk of obesity and T2DM [13,14]. In this context, studies have found that reduced bacterial diversity in diabetic patients is reflected, for example, in a decreased abundance of short-chain fatty acid (SCFA) producers, notably butyrate-producing bacteria (primarily *Roseburia intestinalis* und *Faecalibacterium prausnitzii*) [15,16]. Other studies highlight the significant role of *Akkermansia muciniphila* as a mediator of metabolic benefits (e.g., by positively affecting glucose homeostasis) that are less abundant in prediabetic persons and newly diagnosed T2DM patients [17,18]. For more information on gut microbiota shifts coupled with changes in glycemic status (healthy → T2DM), see the excellent review by Umirah et al. [19]. Another important observation was the increased intestinal permeability in patients with T2DM compared with healthy controls. This “leaky gut” can result in the entrance of inflammatory molecules into the patient’s systemic circulation and consequently lead to increased IR [20,21,22]. The differences in microbiota composition and gut barrier function between healthy individuals and T2DM patients are shown in Figure 1.

This review analyzes the effects of exercise training on the gut microbiota composition and the intestinal barrier function in T2DM. Furthermore, exercise training is considered to be a microbiota/microbiome-based therapeutic approach in T2DM management.

## 2. Influence of Exercise Training on the Gut Microbiota and the Gut Barrier Function in T2DM

The use of data analysis to explore the relationships between physical activity and intestinal colonization patterns is relatively new. Due to advances in sequencing technologies (next-generation sequencing), it is possible to collect several types of data sets. Ribosomal RNA (rRNA) sequencing can help to identify microbial species. Metatranscriptomic data provide a description of active gene expression and the microorganisms’ functional potential [23]. 16S rRNA analysis is the most commonly used method in metagenomics, although shotgun metagenome analysis is bound to replace this technique because it provides higher phylogenetic resolution [24].

Lambert et al. [25] were the first to examine the relationship between T2DM, gut microbiota, and exercise training in 2015. For this purpose, type 2 diabetic *db/db* mice and *db/*^+^ control littermates (diabetic *db/db* mice were obese and had less muscle mass in comparison to non-diabetic *db/*^+^ controls) were randomized to a sedentary or an exercise group for 6 weeks. The exercise training consisted of moderate- to high-intensity treadmill running for 5 days/week. Their results showed that exercise training could affect bacteria abundance/composition. One main effect of exercise was the greater abundance of the Firmicutes species *Clostridium* cluster *C-I* and lower *Bacteroides/Prevotella* spp. and *Methanobrevibacter* spp. in both normal and diabetic exercised mice compared with their sedentary counterparts. *Bifidobacterium* spp. increased in exercised normal mice, but decreased in exercised diabetic mice (exercise × diabetes interaction); all results were adjusted for body weight and blood glucose. Some of the results suggest that changes caused by diabetes can be reversed through exercise training. In this context, for example, increased *Bacteroides*/*Prevotella* spp. has been identified in T2DM patients compared with healthy subjects [26]. Furthermore, their results also indicate that exercise-induced changes in gut bacteria can differ between diabetic and non-diabetic organisms.

More recently, another animal study was conducted and concluded that regular physical activity can positively influence gut bacteria abundance/composition and the microbial SCFA synthesis, thereby ameliorating IR [27]. Diabetic mice trained over 8 weeks (swimming exercise, 5 days/week) showed a more balanced abundance of microorganisms of Firmicutes and Bacteroidetes (Bacteroidetes decreased in diabetic mice and increased through training, with no change in Firmicutes) and decreased IR (glucose tolerance was improved) following exercise training. Furthermore, in this study, regular physical activity upregulated microbial SCFA synthesis, thereby reversing the decline in intestinal and total plasma SCFA concentrations observed in T2DM mice compared with the SCFA concentrations in non-diabetic animals. The following experiment, which was also conducted by the group, underlined the important role of SCFAs in insulin sensitivity. The administration of a G protein-coupled receptor 43 (GPR43) inhibitor diminished the training-mediated improvement of IR. SCFA-sensing GPRs (G protein-coupled receptor 41 (GPR41), GPR43, G protein-coupled receptor 109 (GPR109), and olfactory factor 78) are present in intestinal epithelial cells, adipocytes, and immune cells mediating health-related metabolic processes [28].

Improved health variables associated with gut microbiota changes were likewise observed in human studies when subjects followed specific training programs. Motiani et al. [29] analyzed the effects of exercise training (sprint interval training (SIT) or moderate-intensity continuous training (MICT), 3 times per week for 2 weeks) on gut microbiota and on intestinal insulin-stimulated glucose uptake (GU) in sedentary, insulin-resistant individuals. Microorganisms of the Bacteroidetes phylum increased, whereas the F/B ratio, bacteria of the *Clostridium* and *Blautia* genera (for *Blautia* only by trend) decreased in both training groups. *Clostridium* and *Blautia* have been shown to belong to the most abundant genera in prediabetes and T2DM compared with healthy subjects [23,30]. Species of *Clostridium* play an important role in the whole-body immune response, while species of *Blautia* can increase the release of pro-inflammatory cytokines. In this context, both training interventions reduced the systemic pro-inflammatory tumor necrosis factor α (TNF-α) level in prediabetic or diabetic participants. In relation to metabolic endotoxemia, both training modes tended to reduce both C-reactive protein (CRP) and intestinal inflammatory lipopolysaccharide binding protein (LBP). Additionally, Bacteroidetes correlated negatively with the plasma inflammatory markers LBP, CRP, and TNF-α, highlighting a possible role of Bacteroidetes in the regulation of (intestinal) inflammation in T2DM. Furthermore, at baseline, the insulin-stimulated GU (from circulation) correlated positively with the abundance of Bacteroidetes and inversely with the abundance of Firmicutes, the F/B ratio, and *Blautia* genus. It can thus be assumed that the exercise training-induced changes in gut microbiota could have a positive effect on GU (or vice versa) [29]. However, there was no significant change in insulin-stimulated GU in either training group. In addition to these results, it is also noteworthy that the abundance of the aforementioned *Blautia* genus was inversely correlated with whole-body insulin sensitivity. Motiani et al. [28] also showed that different training regimes can affect gut microorganisms differently. For example, SIT leads to a higher abundance of *Lachnospira* genus whereas MICT raises the abundance of the *Veillonella* genus, both in comparison to baseline values. Furthermore, only MICT was able to increase the abundance of *Faecalibacterium* genus (particularly *F. prausnitzii*).

Comparable results of improved endotoxemia were found by Pasini et al. [31], who examined the effects of chronic exercise on the gut microbiota and intestinal barrier function in T2DM patients. All subjects followed a 6-month program of endurance (cycling at moderate intensity), whole-body resistance, and flexibility training (3 exercise sessions per week), and followed an energy-restricted diet (1900 kcal; consisting of 40–60% carbohydrates, 30% fat, and 10–20% protein). At baseline, T2DM patients had an overgrowth of intestinal mycetes, increased intestinal permeability (measured by elevated fecal zonulin concentration), systemic low-grade inflammation (measured by CRP), and decreased insulin sensitivity (determined via the homeostatic model assessment of insulin resistance (HOMA-IR) index). In addition to mycetes overgrowth, the abundance of *Candida albicans*, a pathogenic biofilm former [32], was increased. After six months of endurance, resistance, and flexibility training, all of the aforementioned outcomes improved, with reduced amounts of mycetes, reduced abundance of *Candida albicans*, and reduced zonulin levels [31]. Moreover, chronic exercise decreased the HOMA-IR index and reduced systemic low-grade inflammation. However, it should be noted that these results should be interpreted with caution because dietary changes resulting in reductions in body weight, body mass index (BMI), fat mass, and waist circumference may also have an impact on the outcomes.

By contrast, Liu et al. [33] reported mixed responses to regular physical activity in subjects with impaired glucose regulation. In the study, prediabetic, medication-naïve, overweight men participated in an exercise intervention. The study included a detailed analysis of the metagenome and the metabolome in feces before and after 12 weeks of training. Participants maintained their habitual diet and were allocated to either a control group or a supervised high-intensity endurance/resistance training program. Overall, the intervention showed metabolic benefits, but nearly 30% of the subjects responded poorly to the combined training mode in terms of ameliorating insulin sensitivity. Subsequently, for further analysis, the exercise training group was divided into “responders” and “non-responders” to exercise training in terms of improvement in insulin sensitivity. There was a clear difference in microbial composition and function between exercise responders and non-responders. Responders showed a decreased abundance of *Bacteroides xylanisolvens* while the *Streptococcus mitis* group increased from pre- to post-training. In addition, responders were characterized by a 3.5-fold higher abundance of *Lachnospiraceae bacterium* (a butyrate producer) and a 43% reduction in *Alistipes shahii* (a possible inflammation promoter [34]). By contrast, non-responders showed a decrease in *Ruminococcus gnavus* and a 3.9-fold increase in the aforementioned *Alistipes shahii.* Regarding the growth dynamics of bacteria, responders showed a decreased replication rate of *Prevotella copri* (a possible contributor to insulin resistance in association with the biosynthesis of branched-chain amino acids (BCAAs) [35]) and an increased growth rate for several species in the *Bacteroides* genus, most of which were propionate synthesizers [36]. The additional microbiome analysis of responders confirmed the changes in gut microbiota and showed greater expression of relevant genes for metabolic pathways synthesizing SCFAs and leading to a breakdown of BCAAs. 

In a second experiment, Liu et al. [33] transplanted the microbiota of two responders and two non-responders from the aforementioned experiment in antibiotic-treated mice to study the potential link between differently compounded microbial gut colonies based on exercise training and changes in glycemic control/insulin sensitivity. The mice that received microbiota from responders showed reductions in glucose and insulin levels, as well as improvements in glucose clearance/disposal during glucose and insulin tolerance tests. Moreover, the mice that were inoculated with gut microbiota from responders exhibited increased levels of circulating SCFAs and decreased concentrations of BCAAs. By contrast, there were no changes in glucose regulation, insulin sensitivity, and SCFAs, nor in BCAAs in mice colonized with the microbiota of non-responders. 

Collectively, these results suggest that exercise training-induced changes in the gut microbiota are (co-)responsible for changes in glycemic control/insulin sensitivity. Furthermore, the intestinal microbiota composition appears to be a determinant for the efficacy of exercise training due to the fact that the baseline microbiome composition also differed between responders and non-responders. Therefore, targeting the gut microbiota could help predict the benefits of exercise. For this reason, the research team developed a model based on an algorithm that can predict the effects of exercise training from baseline microbial signatures. However, it should be noted that this algorithm is a prototype that needs to be refined in further studies.

In addition to the aforementioned positive effects of regular physical activity on glycemic control/insulin sensitivity, exercise training (together with other lifestyle changes) is known to contribute to changes in body composition (increases in muscle mass and reductions in fat mass) [37,38]. It is important to note that a changed body composition can result in a different microbiome composition [39].

In summary, regular physical activity can change the gut microbiota composition in T2DM patients with a higher abundance of SCFA producers [25,29,33]. This increased abundance of SCFA producers leads to elevated intestinal and plasma SCFA concentrations, which are able to ameliorate skeletal muscle IR [27]. Furthermore, exercise training can reduce the abundance of specific microbes (e.g., *Alistipes shaii*, *Prevotella copri*, or microbes of the *Blautia* genus) that contribute to inflammation and IR, thereby ameliorating endotoxemia, whole-body insulin sensitivity, and improving glycemic control [29,31,33] (for an overview of all relevant studies and study details, see Table 1). The special role of exercise and the potential molecular mechanisms that contribute to the alleviation of T2DM are described in detail below.

## 3. Microbial Metabolites and Possible Mechanisms That Contribute to Alleviation of T2DM

Currently, the mechanisms through which exercise training-induced changes in gut microbiota and intestinal permeability occur and positively influence health outcomes in T2DM are not yet fully understood. However, studies suggest that several aspects might explain how regular exercise can influence gut microbiota and gut permeability, thereby promoting positive health outcomes in T2DM.

### 3.1. Exercise and Exercise Training-Induced Production of SCFAs

Following a training intervention, the gut microbiota is able to synthesize several metabolites that are involved in the regulation of the host’s metabolism [40,41]. In this regard, SCFAs (especially butyrate, acetate, and propionate) seem to be the most important microbial metabolites, possibly explaining the positive influence of regular exercise in T2DM [27,40,41]. Usually, these SCFAs result from the microbial fermentation of non-digestible carbohydrates, such as cellulose, pectin, and oligosaccharides [23,40,42]. In 2019, Scheiman et al. [43] showed that a *Veillonella* strain was most likely able to use exercise-induced lactate for the production of SCFAs. *Veillonella atypica* was isolated from a stool sample of marathon runners and transferred to mice. In the subsequent running test to exhaustion, these mice showed improved exercise performance and converted lactate more effectively to propionate in comparison to the control animals colonized with *Lactobacillus bulgaricus*. Moreover, Scheiman et al. [43] found that systemic lactate was able to cross the gut barrier into the lumen, making it available as a substrate for SCFA synthesis by *Veillonella atypica*. The produced SCFAs could subsequently enter the systemic circulation, with potential metabolic effects. 

Yu et al. [44] were also able to show that SCFA production is important for health outcomes. They investigated the effects of a training intervention (moderate-intensity treadmill running, 30–60 min/day for 8 weeks) and/or direct butyrate supplementation on gut microbiota in different animal groups ((1) normal diet; (2) normal diet + exercise training; (3) high-fat diet; (4) high-fat diet + oral sodium butyrate intake; (5) high-fat diet + exercise training; or (6) high-fat diet + exercise training + oral sodium butyrate intake). Both the training intervention and/or butyrate supplementation partly reversed metabolic dysfunction and inflammation (TNF-α, interleukin-1β (IL-1β), lipopolysaccharides (LPS)) generated in mice by a high-fat diet. Eight genera increased through exercise training. Of these eight genera, six were able to synthesize butyrate, including *Enterobacter*, *Bacteroides*, *Roseburia*, *Prevotella*, *Paraprevotella*, and *Akkermansia*. It can be assumed that the butyrate production of these microorganisms could explain the beneficial effects of regular physical activity on systemic low-grade inflammation and metabolic endotoxemia. However, basal butyrate levels in the serum and feces were not significantly increased in high-fat diet mice following physical training in this study. It may be possible that the training stimulus (intensity and/or duration) in this study was not sufficient to increase basal butyrate levels. A transient increase in butyrate following acute exercise could not be excluded (but has not been proven).

By contrast, in the animal study conducted by Yang et al. [27], intestinal and plasma SCFA concentrations were increased through regular physical activity (as mentioned earlier in this review): butyric acid, propionic acid, and acetic acid were all increased in feces and acetate in plasma. The phosphorylation activity of IRS at ^Tyr612^ and AKT at ^Ser473^ in relation to their unphosphorylated state (important for the insulin signaling process) was increased in skeletal muscle cells of trained diabetic mice compared to those of sedentary diabetic mice. The administration of the GPR inhibitor GLPG0974 (background: GPR43 interacts with SCFAs) diminished the exercise training-mediated re-activation of phosphorylation activity in the insulin signaling pathway, indicating the possibly central role of SCFAs in the exercise training-induced improvement of insulin sensitivity. To further corroborate this idea, the roles of the GPR receptor (inhibitor) and SCFAs were explored in the primary muscle cells of wild-type mice. Insulin resistance in the cells was induced with palmitate. Decreased abundances of p-IRS ^Tyr612^ and p-AKT ^Ser473^ were significantly alleviated after SCFA (sodium acetate) treatment. However, the subsequent administration of GPR43 inhibitors reversed the remission effect of sodium acetate. These results confirmed that exercise and the SCFA-mediated improvement of skeletal muscle IR was dependent on the interaction between SCFAs and GPR43, as already mentioned before. 

In summary, these outcomes strengthen the assumption that exercise can induce microbial SCFA production, with SCFAs binding to GPR43 and reversing skeletal muscle IR. Nevertheless, the final and essential biochemical step between SCFA binding to GPR43 and amelioration of skeletal muscle IR is not yet fully understood. Further studies are needed in this area to fully understand the underlying molecular mechanisms.

The following two sections describe further specific functions of SCFAs in the context of exercise training-induced benefits in T2DM.

### 3.2. Exercise Training-Induced Improvement in Gut Barrier Function

Under normal conditions, the gut barrier, which consists of inner and outer mucus layers within the lumen and epithelial cells connected by tight and adherens junctions, prevents bacterial components, such as LPS, from translocating into the underlying lamina propria and systemic circulation [45]. As mentioned before, T2DM is often associated with gut barrier dysfunction, but some studies mentioned above have shown that regular physical activity can improve increased intestinal permeability and endotoxemia in T2DM patients [29,31]. This might be due to the fact that physical activity is probably able to decrease intestinal zonulin concentration, a protein known to destabilize tight junctions and increase intestinal permeability [31,45]. Furthermore, the improvement in gut barrier integrity might be attributable to the exercise training-induced higher abundance of SCFA producers, in particular butyrate producers such as *Faecalibacterium prausnitzii* [29]. Butyrate has been shown to induce the differentiation of colonic regulatory T (T_reg_) cells with the production of anti-inflammatory cytokines (e.g., interleukin-10 (IL-10)) promoting gut barrier function [46]. IL–10–deficient mice have been shown to develop a primary intestinal permeability defect in response to normal enteric microflora [47]. Mechanistically, in a cell culture experiment, Furusawa et al. [46] found that butyrate was able to enhance histone H3 acetylation in the promoter of the Forkhead–Box–Protein P3 (FOXP3) gene (leading to increased FOXP3 expression). FOXP3 is an important transcription factor that is crucial for T_reg_ function. This might be a possible epigenetical mechanism for how exercise training-induced microbial–derived butyrate improves gut barrier function. Nevertheless, the precise mechanism(s) by which the gut barrier, as well as the tight junction, work, and how they are influenced by external factors is/are still not fully understood. Further studies are needed to decode the possible biochemical and epigenetical mechanisms [48]. 

### 3.3. Influence of Exercise Training on Intestinal Transit Time

In general, T2DM patients have a prolonged intestinal transit time (ITT) because elevated glucose levels reduce gastric and small intestine motility during fasting and after food intake [49,50]. A slower ITT leads to increased nutrient absorption and energy harvest from the diet, with possible disturbance of energy homeostasis and the development of adiposity as well as T2DM [49]. One possible impact of regular physical activity is an altered ITT. However, the available data on this aspect are controversial. On the one hand, studies indicate that regular physical activity can shorten the ITT [51]. This shorter ITT seems to be a determinant affecting the gut microbiota with an increased abundance of fast-growing species from the *Ruminococcaceae–Bacteroides* enterotype (most of which are SCFA producers). They have an advantage because of their short replication time. Other microbes with longer replication times run the risk of being washed out [36,52]. The SCFAs are able to interact with GPR41 and GPR43 on intestinal L-cells. L-cells belong to the enteroendocrine cells in the epithelium of the gut, which hormonally regulate digestive and satiation processes [53]. Furthermore, it has been demonstrated, in isolated rat colon, that SCFAs can increase glucagon-like peptide-1 (GLP-1) secretion [54,55]. GLP-1, an incretine hormone, is released after food intake and contributes to increased insulin release from β-cells in a glucose-dependent manner [56]. It might be speculation that the increased production of microbial SCFAs may help ameliorate the reduced effect of incretin, which is often evident in T2DM patients, but further studies are needed to understand this process in more detail [57]. Currently, it is still uncertain how microbiota-produced SCFAs binding to GPR receptors can contribute to an increased GLP-1 secretion [55,58]. 

On the other hand, one study showed that the response to exercise training in relation to ITT can be highly individual. Through exercise over one week (1 h walking at a speed of 4.5 km/h; 3 times/week), ITT was decreased in 5, increased in 6, and did not change in 5 healthy, previously sedentary subjects (total *n* = 16) in a study by Robertson et al. [59]. Therefore, it cannot be excluded that regular physical activity could also contribute to prolonged ITT. The possible effects of regular physical activity on ITT in a pathological state, and in particular in T2DM patients, have not been explicitly investigated in a study. It should also be noted that prolonged ITT might not only have disadvantages, but may also have advantages. It has already been shown that prolonged ITT can increase dietary protein catabolism with higher intestinal indole derivate synthesis [60]. These bacterial-produced indoles are also able to positively influence GLP-1 secretion by intestinal L-cells and some indole derivates, such as indolepropionic acid or indole-3-acetic acid, were even shown to ameliorate IR and low–grade inflammation status in T2DM [40]. It should, however, be noted that excessive colonic protein fermentation (mainly from animal protein sources) favors a potentially pathogenic and pro–inflammatory microbiota profile (higher abundance of *Clostridium* spp. and *Enterococcus* spp.) with increased ammonia, phenols, and hydrogen sulphide concentrations (potentially harmful microbial metabolites) and decreases in the production of SCFAs [60]. These potentially harmful metabolites largely compromise the colonic epithelium structure, causing mucosal inflammation and possibly contributing to the development or progression of T2DM [61,62]. 

In summary, exercise could potentially alter ITT (possibly in both directions). The effects of ITT on the gut microbiota in relation to T2DM have not yet been researched in detail. 

### 3.4. Exercise-Induced Secretion of Myokines

In the exercise training-induced secretion of SCFAs, myokines released within and from contracting skeletal muscle fibers could explain some of the relationships between physical activity, the gut, and T2DM [41]. In particular, interleukin-6 (IL-6) produced by muscle cells seems to have an anti-inflammatory effect in relation to microbial endotoxins. Starkie et al. [63] were able to show that either exercise (3 h of ergometer cycling; intensity 75% of VO_2_max) or an IL-6 infusion significantly suppressed endotoxin-induced pro-inflammatory TNF-α production in healthy subjects (participants were randomized in three groups (rest, bicycle ride for 3 h, or rest and infusion with recombinant human IL-6). 

Based on this result, it can be assumed that a regular exercise-induced release of IL-6 suppressing the endotoxin-induced secretion of pro-inflammatory cytokines may be one explanation for improved endotoxemia in physically active T2DM patients over the long term [29]. 

Another important myokine is irisin. Irisin can convert white adipose tissue into brown adipose tissue, a process known as browning, which results in increased total energy expenditure [64]. Irisin could contribute to the reduction of hyperlipidemia and hyperglycemia in patients with T2DM [65]. Similar to IL-6, irisin concentration can be increased by acute exercise [63]. A study showed that serum irisin can also be increased by exenatide treatment (a GLP-1 receptor agonist) [66]. This leads to the assumption that exercise training-induced changes in the microbiome with increases in SCFA producers can also contribute to increased irisin levels in the long term, as SCFAs are able to induce GLP-1 secretion. This could explain some metabolic benefits of training-induced changes of the microbiome. However, further clarification is needed. 

## 4. General Considerations

As regards the possible positive effects of exercise training on the gut microbiota and the gut barrier integrity in T2DM patients, there are some critical points that may need to be taken into account when interpreting the study results. (1) The interpretation of gut microbiota and microbiota-based individual therapeutic measures is difficult in patients with diabetes, because it is currently not clear whether the changes in gut microbiota composition are the cause or consequence of T2DM [67]. (2) Some conclusions are based on a very limited number of studies with heterogenous study designs. (3) The study populations differ in terms of age, diet, body weight/composition, diabetes complications, and diabetes medication, which can also influence the gut microbiota composition, independent of exercise-induced effects [7,9,11,23,40]. (4) Exercise interventions are highly heterogenous in type (endurance, resistance, or combined training), training frequency (e.g., daily [27] vs. 3 times/week [29,31,33] vs. 5 times/week [25]), training duration (e.g., 60 to 90 min [27,31]) and intensity (e.g., moderate- to high-intensity) [29,33]) and it is therefore difficult to compare their effects. (5) Chronic exercise training as performed in Pasini et al. [31] may reduce body weight and waist circumference, resulting in a reduction of visceral fat. This decreased visceral fat mass leads to the attenuation of pro-inflammatory cytokine release, which may be one mechanism through which chronic exercise improves gut barrier integrity, independent of the direct gut-mediated metabolic reactions [45]. (6) Different microbiota analysis methods are used in the studies (16S rRNA amplicon analysis [27,29] vs. selective agar culture medium [31] vs. real-time quantitative polymerase chain reaction (RT-qPCR) [25,39] vs. whole metagenome shot-gun analysis and fecal metabolomics [33]), which can lead to different results in taxonomic range and strain-level resolution due to the different type of culturing or sequencing, the use of variable reference databases that are highly incomplete, and different opportunities in statistical analysis [24].

Therefore, further studies should pay more attention to standardizing procedures for recruiting subjects, implementing exercise interventions, and analyzing microbiota. 

## 5. Conclusions and Future Directions

**Figure 2:**
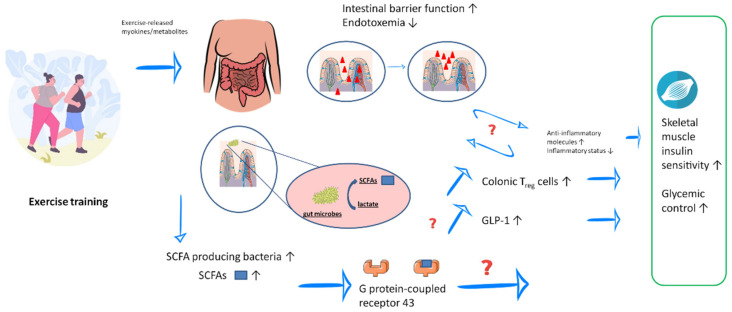
Possible interactions between exercise training, gut health, and type 2 diabetes mellitus. Regular exercise can increase the number of short-chain fatty acid (SCFA)-producing bacteria and SCFA concentrations in the gut and blood. Some microbes may convert lactate released during exercise into propionate. SCFAs may improve inflammatory status as well as insulin sensitivity and glycemic control via G protein-coupled receptor 43 and its downstream pathways. Exercise training can also improve the gut barrier function, leading to reduced endotoxemia. *GLP-1* glucagon-like peptide-1, *SCFA* short-chain fatty acid, *T2DM* type 2 diabetes mellitus, *Treg* regulatory T cells. Graphical sources: https://de.freepik.com/vektoren-kostenlos/satz-von-acht-flachen-runden-kompositionen-zur-gewichtsreduktion-mit-menschen-die-essen-essen-und-fitnessuebungen-machen_21744750.htm#query=Dicker%20mensch&position=5&from_view=search, https://pixabay.com/de/, https://smart.servier.com/smart_image/intestinal-villi/, https://smart.servier.com/smart_image/bacterium-2 (last access to all four websites: 2 July 2021).

The current body of literature about the relationship between physical activity, gut microbiota, and gut barrier function in patients with T2DM is limited. Nevertheless, the study results indicate that regular exercise can modulate the gut microbiota composition and intestinal barrier function, resulting in possible reductions in low-grade systemic inflammation and increases in insulin sensitivity/glycemic control (Figure 2). It should, however, be noted that the mechanisms by which regular exercise influences the gut microbiota are not yet fully understood. 

Further studies are needed to examine the efficacy of different training modes, the roles of myokines, SCFA-producing bacteria, and SCFAs in relevant metabolic pathways. As microbial signatures differ in individuals who respond differently to exercise training programs, one scientific focus in the future could be the development of computer-based methods for the personalized analysis of the gut microbiota in the context of a microbiota/microbiome-based training program.

## Figures and Tables

**Table 1:** Effects of exercise training on the gut microbiota and the gut barrier function in animals/patients with type 2 diabetes mellitus. Overview of included studies.

Author(s), Year, Country	Animal Model Used or Subjects’ Characteristics	Microbiota/ Microbiome Analysis Method	(Exercise) Intervention	Duration	Main Results
ANIMAL STUDIES					
Yang et al., 2020 [27], China	60 C57BI/6 J mice (HFD-induced diabetes)	16S rRNA sequencing SCFA-analysis: gas chromatography	Three groups: -Non-diabetic controls (no training) -Diabetic animals (no training) -Trained diabetic animals Swimming exercise (60 min/day; 5 times/wk)	8 weeks	Exercise training reduced IR and resulted in a more balanced abundance of Firmicutes and Bacteroidetes (abundance of Bacteroidetes was decreased in diabetes and increased through training) Physical activity increased intestinal and plasma SCFA concentrations GLPG0974 abolished exercise-mediated improvement of IR and acetate-mediated reduction of skeletal muscle IR Muscle cell investigation showed that exercise and acetate-mediated improvement of skeletal muscle IR depends on GPR43 (and its interaction with SCFAs)
Lambert et al., 2015 [25], Canada	Diabetic C57BL/KsJ-leprdb/leprdb mice and non-diabetic db/^+^ littermates	RT-qPCR	Three groups: -Non-diabetic animals (no training) -Diabetic animals (no training) -Trained non-diabetic animals -Trained diabetic animals Moderate- to high-intensity treadmill running, 5 times/wk); physical capacity and genotype of mice determined exercise intensity: db/^+^ mice: 60 min/session at a speed of 4.79 m/min (287 m/session), db/db mice: 66 min/session at a speed of 2.87 m/min (189 m/session)	6 weeks	Main effect of exercise, with increased abundance of the Firmicutes species *Clostridium* cluster *C-I* and lower abundance of *Bacteroides/Prevotella* spp. and *Methanobrevibacter* spp. Interaction effect of diabetes × exercise on total bacteria abundance Abundance of *Bifidobacterium* spp. increased in exercised normal mice but decreased in exercised diabetic mice (exercise × diabetes interaction)
HUMAN STUDIES					
Liu et al., 2020 [33], China	Overweight/Obese prediabetic men; *n* = 39 (20–60 years) C57BL/6J mice	Whole metagenome shotgun analysis and fecal metabolomics	Control group: -No exercise Intervention group: -HI(I)T 70 min combined endurance and resistance interval training; 3 times/wk Training sessions divided in: -10 min warm-up -Three 10 min stations of high-intensity (interval) training (treadmill running, ergometer cycling, resistance, and calisthenics exercises), with 3–4 min recovery between stations. -10–15 min cool-down and stretching exercises. -Treadmill running: 3–4 exercise bouts of 2 min running at 85–95% VO_2max_ separated by 30–45 s intervals of active recovery at 50% VO_2max_. -Ergometer cycling: 4–5 45–60 s cycling intervals at 90–95% PPO with 60–75 s active recovery at 30% PPO between intervals. -Resistance/calisthenics exercises: 2–3 sets of high-intensity exercises (squats, kettlebell swings, planks, burpees) with 30 s rest between each set 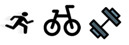	12 weeks	Microbiota composition was differently affected in exercise responders and non-responders from pre- to post-training Exercise responders: decrease in abundance of *Bacteroides xylanisolvens*, increase in abundance of the *Streptococcus mitis* group and *Lachnospiraceae* bacterium, reduction in abundance of *Alistipes shahii*, decrease in replication rate of *Prevotella copri* Exercise non-responders: decrease in abundance of *Ruminococcus gnavus*, increase in abundance of *Alistipes shahii* Exercise training-related gut microbiota changes were associated with improvements in insulin sensitivity The microbiome of responders had increased functional capacity for SCFA synthesis and BCAA breakdown An established learning algorithm was developed to predict individual responses to exercise training based on baseline microbiome Fecal microbiota transplantation from responders in antibiotics-treated mice led to benefits in insulin sensitivity
Motiani et al., 2020 [29], Finland	Overweight/Obese, prediabetic/T2DM men and women; *n* = 26 (49 ± 4 years)	16S rRNA amplicon analysis (V3 and V4 regions)	Two groups -SIT: 30 s exercise bouts (4–6) of all out cycling efforts (4 min recovery); 3 times/wk -MICT: 40–60 min cycling at 60% VO_2 peak_; 3 times/wk 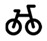	2 weeks	Increased abundance of Bacteroidetes in both groups post-training as well as decreased F/B ratio. Decreased abundance of *Clostridium* and by trend (*p* = 0.051) of *Blautia* genus in both groups post-training Lower abundance of *Blautia* genus was associated with better whole-body insulin sensitivity Effects after SIT: higher abundance of *Lachnospira* genus post- training Effects after MICT: higher abundance of *Veillonella* genus and by trend (*p* = 0.055) of *Faecalibacterium* genus (*F. prausnitzii*) post-training Colonic GU from circulation was positively associated with abundance of Bacteroidetes and inversely with that of Firmicutes phylum, F/B ratio, and abundance of *Blautia* genus
Pasini et al., 2019 [31], Italy	T2DM patients; *n* = 30 (70 ± 2 years)	Stool analysis: Selective agar culture medium	Exercise training: 90 min per session, 3 times/wk + energy-restricted diet Training sessions divided in: -Endurance training 15–35 min cycling, first 3 months: training heart rate 5 bpm below HR_GET,_ last 3 months training heart rate between HR_GET_ and HR_VCP_ -Resistance training: 40–50 min of various exercises (calisthenics, exercises with equipment) involving major muscle groups, 3 sets of 8–15 repetitions, progressively increased -Flexibility training: Static stretching exercises 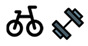	6 months	Exercise training reduced mycetes overgrowth Decreased abundance of *Candida albicans* and reduced zonulin concentration following chronic exercise Improvements in insulin sensitivity and chronic low-grade inflammation (CRP) post-training

BCAA = branched-chain amino acids. bpm = beats per minute. CRP = C-reactive protein. d = day. ELISA = enzyme-linked immunosorbent assay. F/B ratio = Firmicutes/Bacteroidetes ratio. FTI = fat tissue index. GPR 43 = G protein-coupled receptor 43. GU = glucose uptake. HFD = high-fat diet. HI(I)T = high-intensity (interval) training. HOMA-IR index = homeostatic model assessment of insulin resistance index. HR_GET_ = heart rate gas exchange threshold. HR_max_ = maximal heart rate. HR_VCP_ = heart rate ventilatory compensation point. IR = insulin resistance. MICT = moderate-intensity continuous training. min = minutes. RT-qPCR = real-time quantitative polymerase chain reaction. rRNA = ribosomal RNA. SCFA = short-chain fatty acid. SIT = sprint interval training. T2DM = type 2 diabetes mellitus. VO_2max_ = maximal oxygen uptake. VO_2peak_ = peak oxygen uptake. wk = week.

## Data Availability

Not applicable.

## Supplementary Materials

The following supporting information can be downloaded at: https://www.mdpi.com/article/10.3390/nu14040813/s1, Supplemental Material Figure S1. Overview of bacterial taxa. Modified according to [67].
