# Supplementary figures and images for: Exercise for the Diabetic Gut—Potential Health Effects and Underlying Mechanisms
Source: Nutrients. 2022 Feb 15;14(4):813. doi: 10.3390/nu14040813 (PMC8877907; doi:10.3390/nu14040813)

Supplemental Material Figure S1. Overview of bacterial taxa. Modified according to [67].

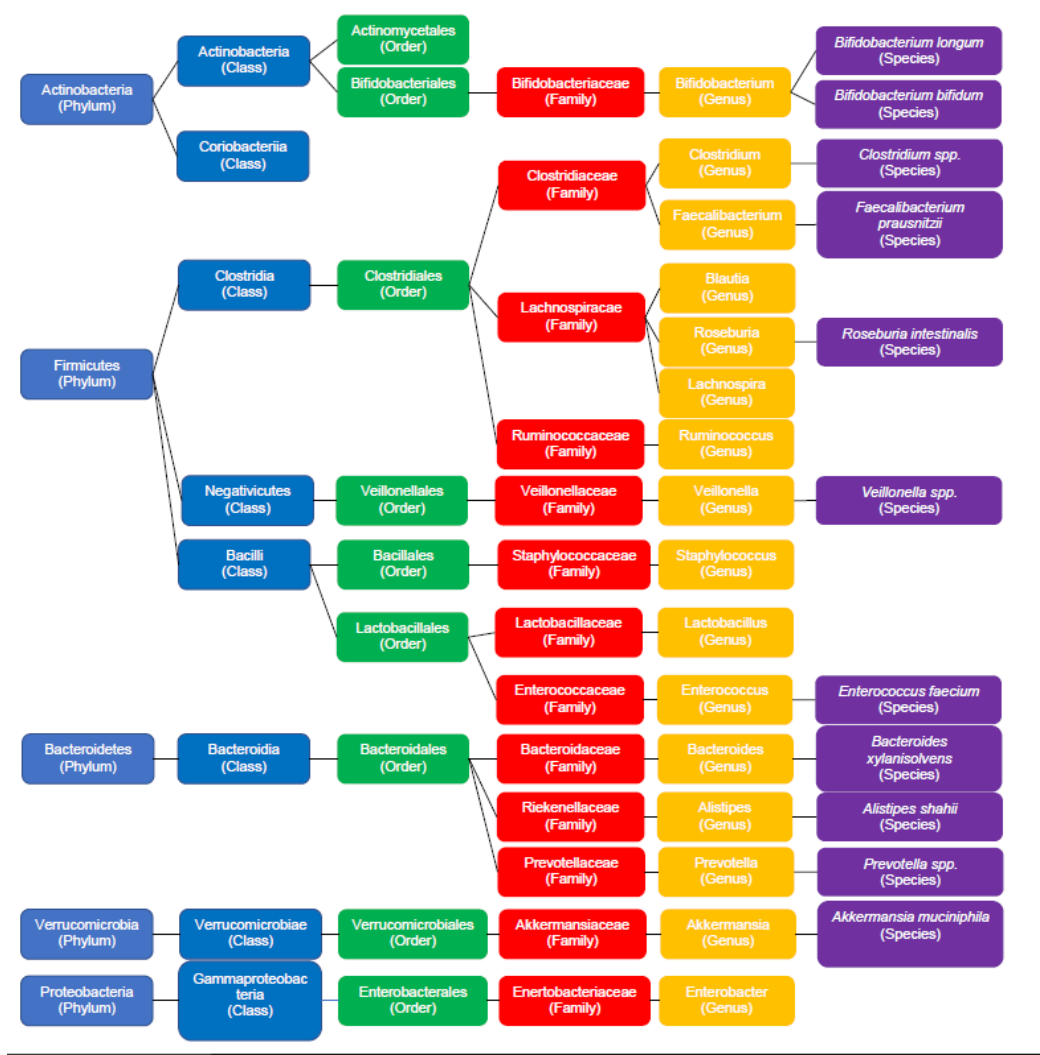

Supplement: Supplementary file 1 [file nutrients-14-00813-s001.zip › nutrients-1577536-supplementary.pdf]
